# Increased doublecortin (DCX) expression and incidence of DCX-immunoreactive multipolar cells in the subventricular zone-olfactory bulb system of suicides

**DOI:** 10.3389/fnana.2015.00074

**Published:** 2015-06-01

**Authors:** Marissa E. Maheu, Julia Devorak, Alexander Freibauer, Maria Antonietta Davoli, Gustavo Turecki, Naguib Mechawar

**Affiliations:** ^1^McGill Group for Suicide Studies, Douglas Mental Health University InstituteMontreal, QC, Canada; ^2^Integrated Program in Neuroscience, McGill UniversityMontreal, QC, Canada; ^3^Department of Psychiatry, McGill UniversityMontreal, QC, Canada

**Keywords:** depression, antidepressants, human postmortem, olfactory bulb, adult neurogenesis

## Abstract

Postmortem studies have confirmed the occurrence of adult hippocampal neurogenesis in humans and implicated this process in antidepressant response, yet neurogenesis in other regions remains to be examined in the context of depression. Here we assess the extent of subventricular zone-olfactory bulb (SVZ-OB) neurogenesis in adult humans having died by suicide. Protein expression of proliferative and neurogenic markers Sox2, proliferating cell nuclear antigen, and doublecortin (DCX) were examined in postmortem SVZ and OB samples from depressed suicides and matched sudden-death controls. In the SVZ, DCX-immunoreactive (IR) cells displayed phenotypes typical of progenitors, whereas in the olfactory tract (OT), they were multipolar with variable size and morphologies suggestive of differentiating cells. DCX expression was significantly increased in the OB of suicides, whereas SVZ DCX expression was higher among unmedicated, but not antidepressant-treated, suicides. Although very few DCX-IR cells were present in the control OT, they were considerably more common in suicides and correlated with OB DCX levels. Suicides also displayed higher DCX-IR process volumes. These results support the notion that OB neurogenesis is minimal in adult humans. They further raise the possibility that the differentiation and migration of SVZ-derived neuroblasts may be altered in unmedicated suicides, leading to an accumulation of ectopically differentiating cells in the OT. Normal SVZ DCX expression among suicides receiving antidepressants suggests a potentially novel mode of action of antidepressant medication. Given the modest group sizes and rarity of DCX-IR cells assessed here, a larger-scale characterization will be required before firm conclusions can be made regarding the identity of these cells.

## Introduction

Adult neurogenesis is a process involving the asymmetric division of neural progenitors to produce neuroblasts which then migrate to discrete brain regions, differentiate into neurons, and integrate into existing circuits. It is now well established that hippocampal neurogenesis occurs in the mature brain of all mammalian species, including humans (Eriksson et al., 1996; Knoth et al., 2010). The other brain region that displays unequivocal neurogenic potential throughout life is the olfactory bulb (OB). The OB receives the majority of its new neurons from the subventricular zone (SVZ) of the lateral ventricles via the rostral migratory stream (RMS), though proliferation continues throughout the RMS and into the OB (Lledo and Saghatelyan, 2005; Whitman and Greer, 2009). The extent to which neurogenesis occurs within the adult human OB, however, remains controversial. Identifying new neurons in the human forebrain has depended largely on the expression of neurogenesis-related proteins, including stem cell markers [e.g., Nestin, sex determining region Y-box 2 (Sox2)], proliferative markers [e.g., proliferating cell nuclear antigen (PCNA), Ki67], and immature neuronal markers [e.g., DCX, polysialylated neural cell adhesion molecule (PSA-NCAM)]. The majority of the evidence in support of continued neurogenesis in the adult human SVZ, RMS, and OB is, therefore, indirect. In 2004, immunolabeling of postmortem human forebrain for neurogenic markers revealed what appeared to be proliferative cells and immature neurons in the granule cell layer of the OB, as well as within the medial olfactory tract (OT; Bedard and Parent, 2004). However, the presence of an RMS in humans has been contested (Curtis et al., 2007; Kam et al., 2009; Sanai et al., 2011), and the rate of OB neurogenesis appears to decay rapidly after birth (Sanai et al., 2011; Wang et al., 2011). As a result, and in contrast to rodents whose OB depend on a constant stream of new neurons to maintain its structural integrity (Imayoshi et al., 2008), fewer than 1% of human OB neurons are likely to be adult-born (Bergmann et al., 2012). Furthermore, recent reports indicate that SVZ-derived neuroblasts may instead migrate toward the human striatum, raising the possibility that variable rates of SVZ neurogenesis may not result in any change in the number of new neurons that reach the OB (Ernst et al., 2014). Given these data, it is unclear how, and how many, adult-born neurons successfully migrate to the OB and contribute to olfactory processing. Moreover, it remains to be determined whether disruptions in this phenomenon accompany the olfactory deficits commonly reported in psychiatric disorders (Pause et al., 2001; Atanasova et al., 2008; Negoias et al., 2010; Nguyen et al., 2010; Sookoian et al., 2011; Zucco and Bollini, 2011; Hardy et al., 2012; Naudin et al., 2012). These impairments include depression-related reductions in odor detection and discrimination (Pause et al., 2001; Atanasova et al., 2008; Negoias et al., 2010), as well as reduced odor processing (Pause et al., 2003).

Several lines of evidence, derived from both animal experimentation and human postmortem studies, implicate altered neuroplasticity in depressive symptomatology. In particular, altered rates of adult hippocampal neurogenesis have received considerable attention as a candidate mechanisms in the expression of depressive behavior and antidepressant action (Perera et al., 2007; Boldrini et al., 2009; Mahar et al., 2011). Conversely, most studies of animal models have found no significant influence of stress or antidepressant treatment (ADT) on SVZ/OB neurogenesis, though results have been inconsistent (Hitoshi et al., 2007). There have been reports of decreased SVZ/OB neurogenesis in rodents exposed to chronic unpredictable mild stress or treated with antidepressants (Mineur et al., 2007; Ohira and Miyakawa, 2011; Yang et al., 2011).

In the present study, we sought to assess OB neurogenesis in well-characterized postmortem SVZ and OB samples from suicides (S) who died during a depressive episode and matched psychiatrically healthy controls (CTRL). Immunoblotting was used to quantify the stem cell marker Sox2, the proliferative marker PCNA, and the migratory neuroblast marker doublecortin (DCX) in these brain regions. In addition, we counted and characterized the morphometric features of DCX-expressing cells in the OT. Our results are in agreement with previous studies indicating minimal OB neurogenesis in adult humans, and suggest that neuroblast migration may be impaired in suicides.

## Materials and Methods

### Subjects

**FIGURE 1:**
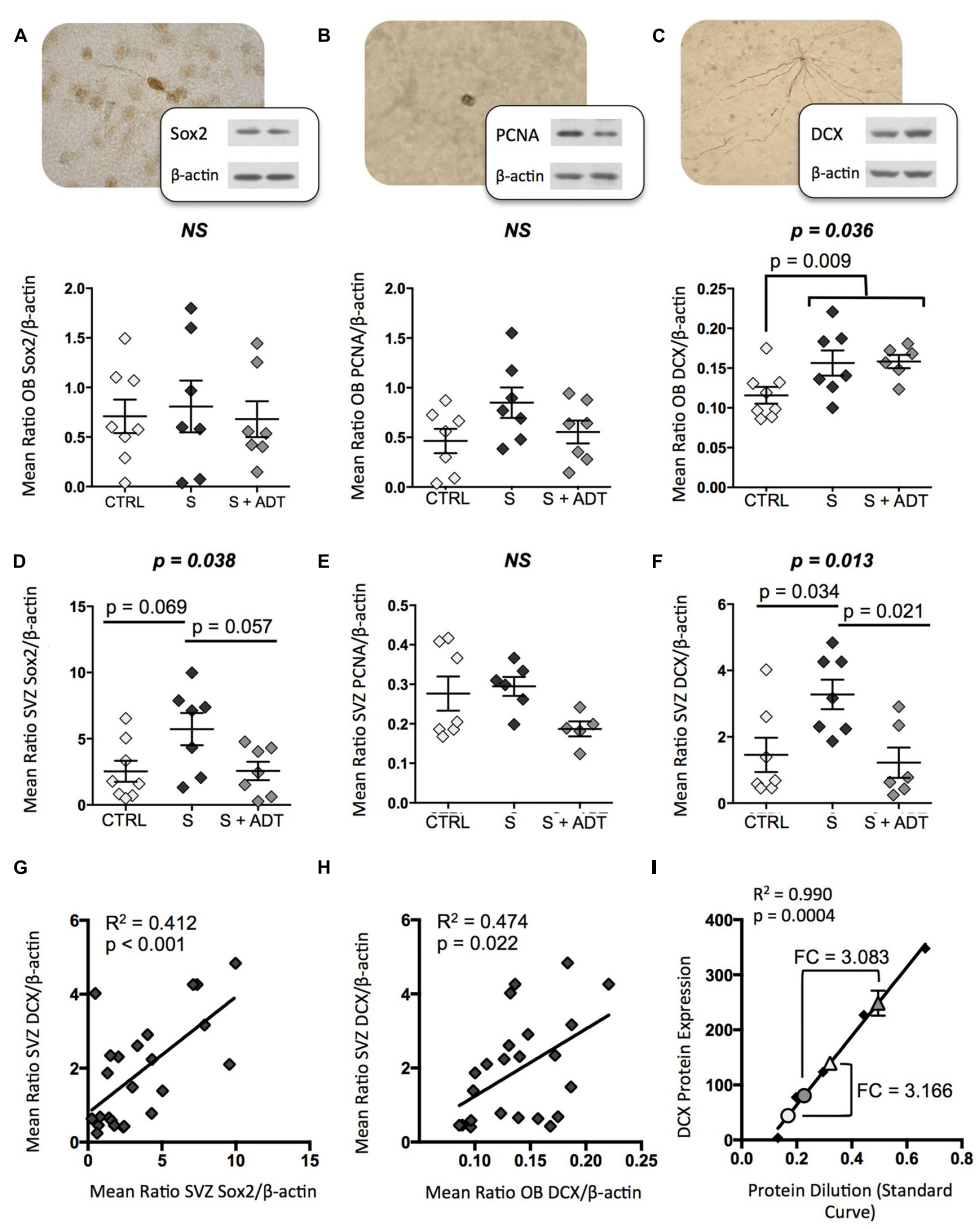
**Expression of neurogenic markers Sox2, proliferating cell nuclear antigen (PCNA), and doublecortin (DCX) in the olfactory bulb (OB) and subventricular zone (SVZ) of suicides (S; *n* = 7), antidepressant treated suicides (S + ADT; *n* = 7), and matched controls (CTRL; *n* = 8). (A,B)** Medicated and unmedicated suicides did not differ from controls with regards to OB Sox2 expression or PCNA levels. **(C)** The S group displayed significantly higher DCX expression than did CTRLs (*p* = 0.009). **(D)** ADT did not reverse the increased DCX expression observed in the OB of S subjects. In the SVZ, although groups did not differ with regards to PCNA expression, unmedicated suicides displayed a tendency toward higher Sox2 levels than both CTRLs (*p* = 0.069) and medicated suicides (*p* = 0.057). **(E)** SVZ PCNA expression did not differ between groups. **(F)** DCX levels were significantly higher in unmedicated suicides than in CTRLs (*p* = 0.034) or medicated suicides (*p* = 0.021). **(G,H)** Sox2 levels were positively and significantly correlated with DCX expression in the SVZ (*p* < 0.001), as were SVZ and OB DCX levels (*p* = 0.022). **(I)** Comparisons of pooled SVZ (triangles) and OB (circles) samples from controls (white fill) and suicides (gray fill) using a standard curve of known protein dilutions revealed that DCX expression in the OB was approximately one third that of the SVZ in both groups. FC, fold change. All data are plotted as means, and error bars depict SEM.

This study was conducted with the approval of the Douglas Hospital Research Ethics Board, and with written informed consent from next of kin. Postmortem brain samples from individuals who died by suicide (*n* = 16) and matched, psychiatrically healthy controls (*n* = 11) were obtained from the Suicide section of the Douglas-Bell Canada Brain Bank (www.douglasbrainbank.ca). Fresh-frozen samples of SVZ containing the anterior horn of the left lateral ventricle and corresponding to plates 7–12 of the Atlas of the Human Brain (Mai et al., 2007) were dissected and collected along with the whole left OB and, when possible, OT (Supplementary Figure S1). Fixed OB from the right hemisphere was collected for Nissl-staining. Due to the small size and rarity of OB and OT tissues, tissues from additional subjects who did not meet criteria for inclusion in group comparisons were employed exclusively to produce representative images of OB staining (**Figures 1A–C**).

Cause of death was ascertained by the Quebec Coroner’s Office. Presence or absence of all medications, drugs of abuse, and alcohol was determined by postmortem blood toxicology. Postmortem psychological autopsies were completed for each subject as described previously (Dumais et al., 2005). Briefly, a trained interviewer completed and the Structured Clinical Interview for DSM-IV Psychiatric Disorders (SCID-I) in consultation with one or more informants known to the deceased. SCID-I assessment, Coroner’s notes, medical records, and case reports were then reviewed by a panel of clinicians in order to obtain a consensus diagnosis. Biological and clinical variables for controls and suicides are presented in **Table 1**. Suicide and control groups did not differ significantly with regards to age [*t*(25) = 0.197, *p* = 0.845], postmortem interval [*t*(25) = 0.547, *p* = 0.589], or brain pH [*t*(25) = −0.049, *p* = 0.962], nor did ANCOVAs reveal significant associations between these factors and dependent variables. Suicides died in the context of a depressive episode, whereas controls died suddenly without evidence of prior or current psychiatric or neurological illness. For group comparisons in which suicides were separated into medicated and unmedicated groups, presence or absence of medications was determined by postmortem toxicology reports provided by the coroner’s office.

**Table 1:** Subject information.

	Controls (CTRL)	Suicides (S)
*N*	11	16
Age (years ± SD)	52 (±16)	52 (±15)
PMI (hours ± SD)	25 (±19)	20 (±25)
Brain pH (±SD)	6.48 (±0.27)	6.48 (±0.31)
Sex (M/F)	7/4	15/1
Axis 1 disorders (*n*)	Nil (11)	MDD (7) D-NOS (4) Schizophrenia (2) Psychotic disorder NOS (1) OCD (1) Bipolar disorder (1) Substance dependence (1)
Cause of death (*n*)	Natural Causes (5) Car accident (3) Cardiovascular (2) Accident (1)	Hanging (10) Intoxication (5) Asphyxia (1)
Medication (*n*)	Nil (11)	Nil (9) Citalopram (SSRI) (1) Amitriptyline (TCA) + Nortriptyline (TCA) (1) Citalopram (SSRI) + Lorazepam (Benzo) + Diclofenac (NSAID) (1) Desmethylvenlafaxine (SNRI) + Venlafaxine (SNRI) + Mirtazapine (TeCA) (1) Desmethyltrimipramine (TCA) + Trimipramine (TCA) + Procyclidine (ACH) (1) Desmethylvenlafaxine (SNRI) + Venlafaxine (SNRI) + Oxazepam (BZD) + Temazepam (Benzo) (1) Desmethylvenlafaxine (SNRI) + Venlafaxine (SNRI) + Clonazepam (BZD) + Methadone (Opiate) (1)
Alcohol present at time of death (*n*)	2	5
Recreational drugs present at time of death (*n*)	THC (1)	Cocaine (2) THC (1) Cocaine + THC (1)

*BZD, benzodiazepine; oxD-NOS, depression not otherwise specified; MDD, major depressive disorder; OCD, obsessive compulsive disorder; NSAID, non-steroidal anti-inflammatory drug; PMI, postmortem interval; SNRI, serotonin-norepinephrine reuptake inhibitor; SSRI, selective serotonin reuptake inhibitor; TCA, tricyclic antidepressant; TeCA, tetracyclic antidepressant; THC, tetrahydrocannabinol*.

### Immunoblotting

Frozen samples were lysed in RIPA buffer containing protease and phosphatase inhibitors (Sigma–Aldrich, St. Louis, MO, USA) and homogenized by sonication. Total protein was extracted and its concentration assessed using the BCA Protein Assay kit (Pierce, Rochford, IL, USA). Protein samples were then resolved through SDS-PAGE and transferred to nitrocellulose membranes to assess expression of neurogenic markers (Sox2, PCNA, and DCX). The specificity of antibodies was confirmed by use of molecular weight markers (Precision Plus Protein WesternC Standards, Bio-Red Laboratories Inc, Hercules, CA, USA) and negative controls (primary antibody omitted). Nitrocellulose membranes were blocked at room temperature in 5% skim milk solution containing 3% normal serum (NS) from the secondary antibody host species. Blots were then treated with the Avidin–Biotin Blocking kit (Vector Laboratories, Burlingame, CA, USA), and incubated overnight in primary antibody solution containing antibody raised against Sox2 (1:500; EMD Millipore, Billerica, MA, USA), PCNA (1:5000; Sigma–Aldrich, St. Louis, MO, USA), or DCX (1:1000; Cell Signaling Technology, Inc., Danvers, MA, USA). Membranes were incubated in a secondary antibody solution containing biotinylated secondary antibody (1:2000; Vector Laboratories, Burlingame, CA, USA) followed by incubation in ABC (Vectastain Elite ABC kit, Vector Laboratories, Burlingame, CA, USA), and the signal revealed with ECL against film. Optical density of target bands was assessed using the MCID system (InterFocus Imaging Ltd., Cambridge, UK), protein expression for each sample normalized using an endogenous control (β-actin), and inter-blot comparisons normalized using pooled calibrators run in triplicate on each gel.

### DCX Immunohistochemistry of SVZ, OT, and OB Tissues

Brain samples were fixed in 10% formalin for 90–120 min at room temperature, then transferred into 15 and 30% sucrose solutions for 24 h at 4∘C prior to being serially sectioned at 50 μm on a SM2000 R freezing microtome (Leica Microsystems, Concord, ON, Canada). Tissue sections were then processed for free-floating immunohistochemistry to label cells expressing Sox2, PCNA, or DCX. In brief, sections were incubated in 0.9% H_2_O_2_ and blocked overnight at room temperature in PBS containing 0.1% Triton and 3% NS. Sections were then incubated in PBS containing 3% NS and one of the primary antibodies described above (1:500 Sox2; 1:1000 PCNA; 1:1000 DCX) for 48–96 h at 4∘C. After washing, tissues were incubated in PBS containing 3% NS and secondary antibody at a concentration of 1:200, followed by incubation in ABC and development with DAB (Vector Laboratories, Burlingame, CA, USA). Sections were then mounted on slides, dehydrated, and coverslipped with Permount. Contrast and exposure have been modified on some of the presented images to improve visibility of cellular structures.

### DCX-Immunoreactive Cell Counting and Reconstruction

All DCX-immunoreactive (IR) multipolar cells present in the OT of S (*n* = 5) and CTRL (*n* = 5) individuals were counted using a Leitz microscope and 40x (NA 0.75) objective, and data expressed as absolute counts of cells and process bundles. Bundles were defined as groupings of DCX-IR processes within a radius of 1 mm regardless of whether a clearly labeled soma was present. This radius was chosen because it was ∼1.5x the maximum radius of reconstructed DCX-IR cells (whose processes did not extend farther than 750 μm from the soma). Due to the scarcity of DCX-IR cells and processes, individual bundles were easily identifiable.

Reconstructions of DCX-IR cells in three dimensions was completed for all clearly stained and undamaged DCX-IR cells possessing an identifiable soma present in the OT of suicides and controls used for DCX-IR cell counting (*n* = 25 cells). Tracing was completed by an experimenter blinded to group using a 100x (NA 1.40) oil immersion objective and an Olympus BX51 microscope equipped with a motorized stage, a CX-9000 camera, and Neurolucida software (MBF Bioscience, Williston, VT, USA). Processes extended by DCX-IR cells were reconstructed in three dimensions, and their volume, structure, and orientations characterized using Sholl and Fan-In projection analyses, as previously described (Glaser and McMullen, 1984; Torres-Platas et al., 2011).

### Statistical Analyses

Statistical analyses were performed using PASW Statistics 18 (Statistical Product and Service Solutions, Chicago, IL, USA). Graphs were produced using Prism GraphPad v.6 (GraphPad Software Inc., La Jolla, CA, USA), and all measures were expressed as mean ± SD. Shapiro–Wilk and Levene’s tests were employed to assess data normality and equality of variance, and outlier detection for immunoblotting data completed using the 1.5IQR method. For parametric data, *t*-tests were used for two-group comparisons, and one-way ANOVAs with Tukey’s *post hoc* tests were employed for three-group comparisons. When data were non-parametric, Mann–Whitney *U* or Kruskal–Wallis tests were employed instead, and *post hoc* comparisons of three group non-parametric data were completed using Mann–Whitney *U* tests (and in such cases, significance was set at *p* = 0.025 to compensate for multiple comparisons). DCX-IR cell and process counting data were analyzed with two-way ANOVAs, with group as the between subjects factor and OT subregion as the within subjects factor. All tests were two-tailed and all correlations were pairwise.

## Results

### Protein Expression in the SVZ and OB

Immunoblotting revealed that while OB expression of Sox2 and PCNA did not differ between CTRL and S groups, DCX levels were significantly higher in suicides than in controls [*t*(19) = −2.916, *p* = 0.009]. When the suicide group was subdivided into unmedicated versus antidepressant-treated subjects, and DCX expression in these groups assessed along with controls using ANOVA, DCX expression was found to be increased in both medicated and unmedicated suicides [*F*(2,20) = 4.036, *p* = 0.036; **Figure 1C**]. Despite the increase in DCX expression among suicides, however, a preliminary examination of neuronal, glial, and endothelial cell density (Hercher et al., 2009b; Garcia-Amado and Prensa, 2012) in the OB granule cell layer revealed no group differences (Supplementary Figure S2).

Conversely, in the SVZ, none of the proteins quantified were found to differ between CTRL and S subjects as a whole. However, when suicides were again subdivided into medicated and unmedicated subjects, both Sox2 [*F*(2,21) = 3.890, *p* = 0.038] and DCX [*F*(2,19) = 5.619, *p* = 0.013] expression levels differed significantly between groups (**Figures 1D–F**). Whereas pharmacological ADT appeared to have no effect on OB DCX expression, in the SVZ antidepressants were associated with normal DCX levels. As a result, DCX expression was higher among unmedicated subjects than both controls (*p* = 0.034, respectively) and medicated suicides (*p* = 0.021). Likewise, the trend toward increased SVZ Sox2 expression among unmedicated suicides was absent in the treated suicide group.

SVZ Sox2 levels correlated positively with SVZ DCX expression (*R*^2^ = 0.412, *p* < 0.001), as did DCX levels in the SVZ and OB (*R*^2^ = 0.474, *p* = 0.022) (**Figures 1G,H**). DCX levels were approximately threefold higher in the SVZ than in the OB in both groups [*t*(4) = 3.042, *p* = 0.038; **Figure 1I**]. This was confirmed using a pooled sample that included protein from, among others, all subjects used for group comparisons [*t*(4) = 8.363, *p* = 0.001; Supplementary Figure S3]. Finally, due to limitations associated with the acquisition of such human brain tissues, our depressed suicide group contained individuals with a range of psychiatric disorders (**Table 1**). Although no clear pattern emerged with regards to DCX expression among subjects with a primary diagnosis of depression as compared to those with some other Axis I disorder (Supplementary Figure S4), it is impossible to confirm with certainty whether additional group differences would emerge with larger sample sizes. Nonetheless, the present data suggest that increased DCX expression in the OB of depressed suicides as a whole, and in the SVZ of unmedicated depressed suicides, is present regardless of other primary diagnoses.

### DCX-Immunoreactive Cells in the SVZ and OT

**FIGURE 2:**
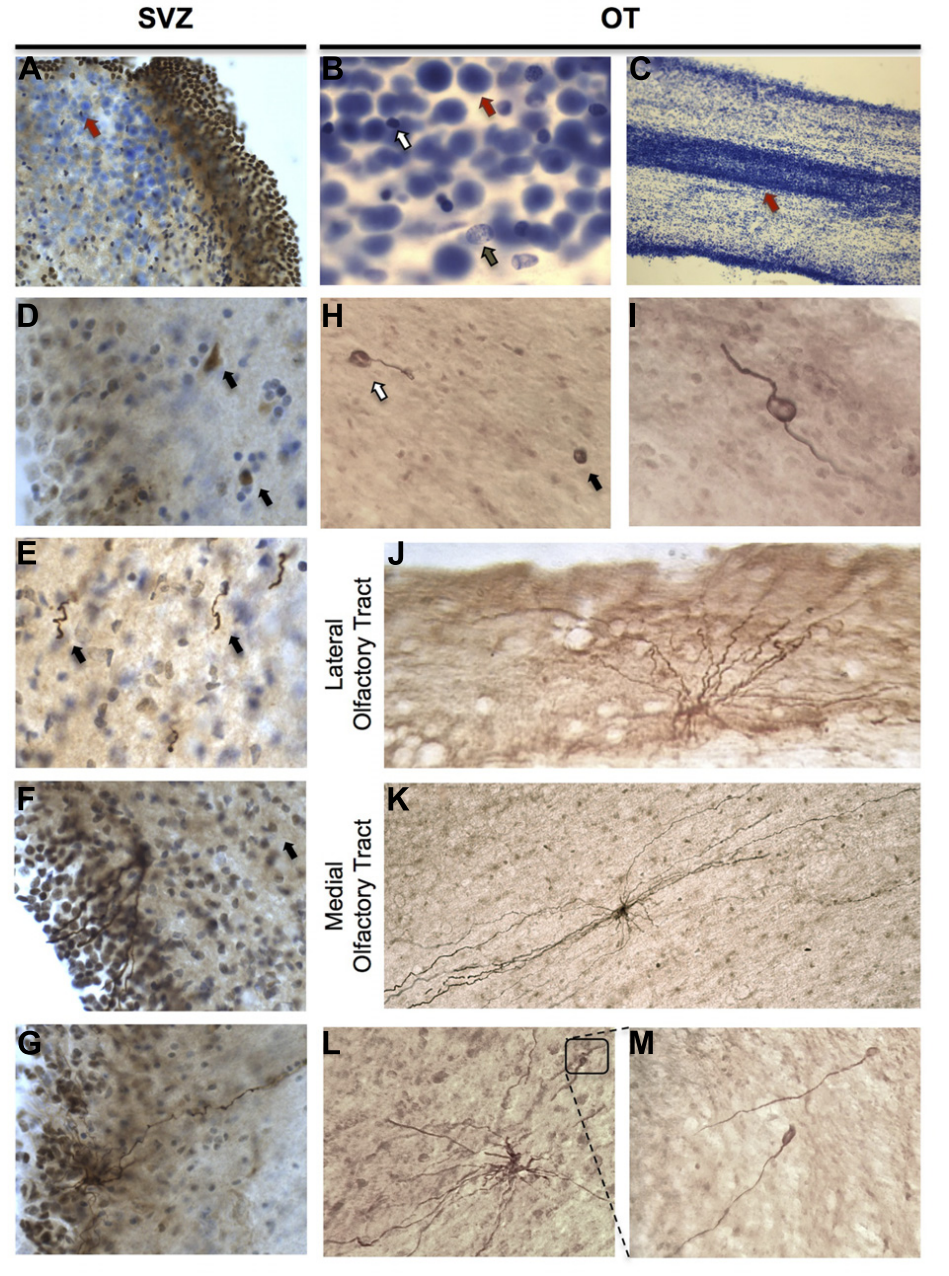
**Cellular composition and characterization of DCX-immunoreactive (IR) cells in the human olfactory tract (OT) and SVZ. (A–C)** Large, densely packed cells of unknown identity were identifiable in SVZ and OT regions and formed a corridor suggestive of a rostral migratory stream. When stained with cresyl violet, these cells (red arrows) were readily distinguishable from both glia (white arrow) and neurons (brown arrow). **(D–G)** DCX-IR expressing cells in the human SVZ displayed variable morphologies including: neuroblast-like cells lacking processes (arrows in **D**); DCX-IR cells with leading and/or trailing processes possessing a rostrocaudal orientation (arrows in **E**); and multipolar cells possessing multiple processes that extended toward the ventricular surface and, occasionally, a additional basal process which projected in the opposite direction. **(H,I)** In the OT, DCX-IR somata with (white arrow) and without (black arrow) processes were observed, as were DCX-IR bipolar cells. **(J,K)** DCX-IR multipolar cells were present in both lateral and medial regions. DCX-IR processes were generally aligned parallel to the tract, particularly in medial OT regions. **(L,M)** Occasionally, DCX-IR multipolar cells possessed bulbous, growth cone-like tips.

In the SVZ, cells displaying three distinct cellular phenotypes were found to express DCX (**Figures 2A–E**). The first subpopulation possessed morphologies consistent with early neuroblasts, displaying DCX-IR somata without processes (**Figure 2D**). Proximal to, but extending beyond, the SVZ, DCX-IR cells displaying a rostrocaudal orientation with leading and/or trailing processes were also observed (**Figure 2E**). Finally, DCX-IR multipolar cells in the SVZ possessed multiple processes that extended toward the ventricular surface (**Figures 2F,G**). Occasionally, these cells displayed a single, long apical process that extended laterally, away from the ventricle, similar to previously published descriptions of SVZ neural stem cells (Mirzadeh et al., 2008).

Similar to the SVZ, DCX-IR cells in the OT displayed varying phenotypes including DCX-IR somata without processes (**Figure 2H**), DCX-IR cells with one process (**Figure 2H**), and cells possessing a bipolar phenotype (**Figure 2I**). Clearly labeled bipolar cells were only rarely observed, however, which is in keeping with previously published reports (Bedard and Parent, 2004). Due to the variable quality of the tissues employed for IHC, and the difficulty of distinguishing small DCX-IR somata lacking processes from background that occasionally possessed a speckled appearance, we did not attempt to quantify the number of DCX-IR somata lacking processes present in the OT. We thus chose to limit our investigation to the characterization of clearly labeled DCX-IR multipolar cells.

### DCX-Immunoreactive Cell Morphology

Reconstructed DCX-IR cells in the human OT varied considerably in size and displayed complex, multipolar morphologies rather than the round or bipolar structure typically associated with migrating neuroblasts (**Figures 2J,K**). Surprisingly, DCX-IR multipolar cells were not restricted to medial regions of the OT, but seemed to be distributed randomly. Rarely, DCX-IR cells were found at the very edge of the tract (**Figure 2J**). In addition, some DCX-IR processes possessed bulbous terminal ends (**Figures 2L,M**), a feature commonly observed on DCX-expressing neurons in the adult human hippocampus (unpublished observation). In the OT these structures were fairly rare, however, appearing only once or twice on a minority of cells.

**FIGURE 3:**
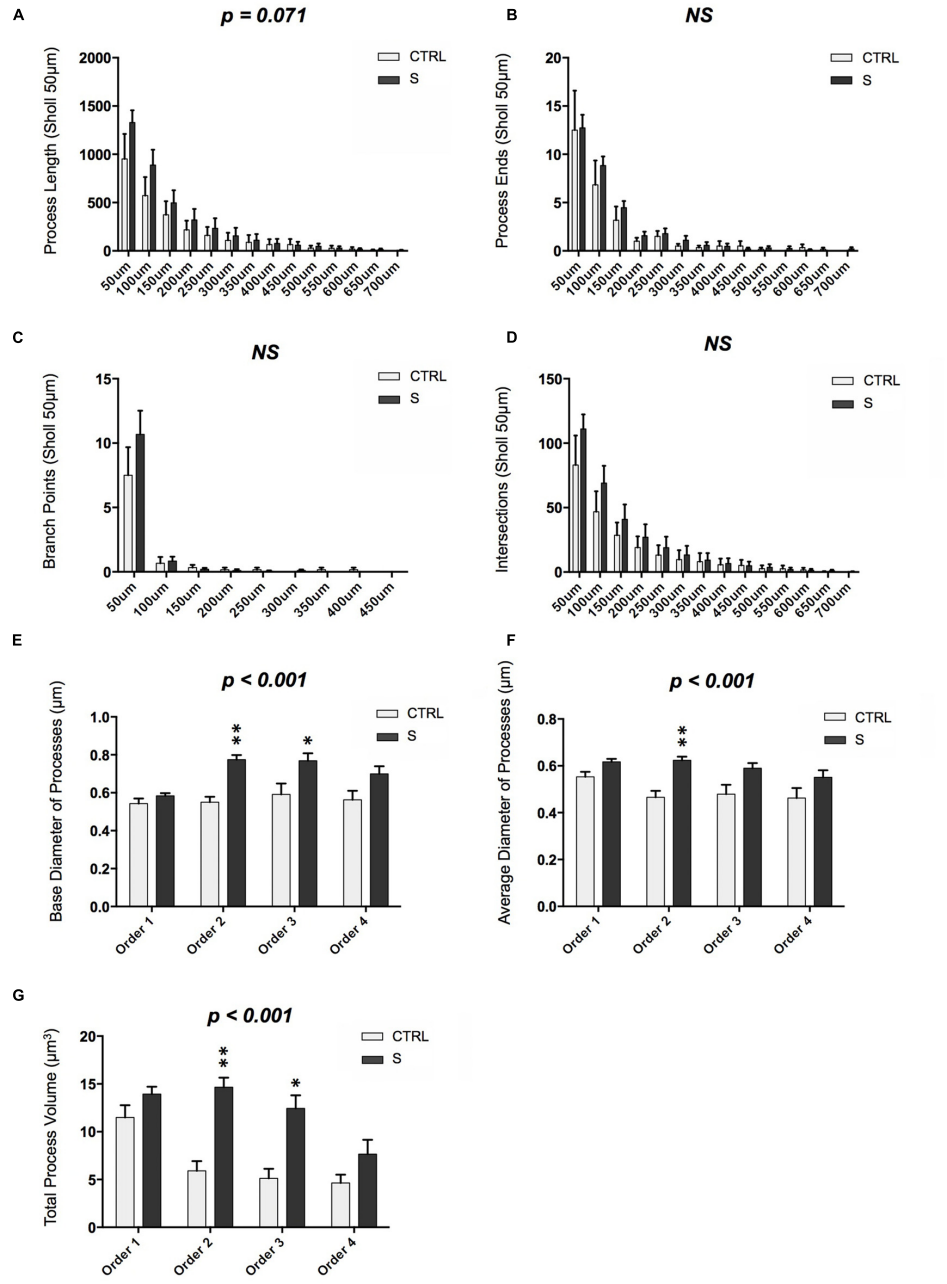
**Morphometric analysis of DCX-IR cells in the OT of suicides (S; *n* = 6 cells) and controls (CTRL; *n* = 19 cells). (A)** DCX-IR cells in suicides showed a tendency toward increased process length as compared to CTRLs (*p* = 0.071). **(B–D)** However, groups did not differ with regards to the number of process ends, branch points, or processes/Sholl intersections. **(E,F)** DCX-IR processes in suicides displayed significantly greater base and average diameter as compared to CTRLs (*p* < 0.001). **(G)** Process volume was also greater among suicides than controls (*p* < 0.001). These increases were due principally to the increased size of second and third order processes. *P*-values above graphs represent significance of “group” main effects. ^∗^*p* < 0.05, ^∗∗^*p* < 0.005. All data are plotted as means, and error bars depict SEM.

Multipolar DCX-IR cells in the OT shared similar morphologies despite being heterogeneous in size (Supplementary Table S1). Larger DCX-IR cells displayed more process branching and complexity, as compared to smaller cells, yet the overall branching pattern did not change. Over 60% of branching events occurred near somata, within a distance equivalent to 20% of the soma’s radius. This morphology was observed regardless of psychiatric diagnosis, with the exception of a trend toward increased process length in suicides [*F*(1,322) = 3.292, *p* = 0.071; **Figure 3A**]. Overall cellular structure of DCX-IR cells, including number of processes, branching points, and process endings, did not differ between groups (**Figures 3B–D**). Similarly, the tortuosity of processes—defined as the length of a process divided by the absolute distance separating the origin and end of that process—did not vary by branch order or between groups [*F*(1,910) = 0.427, *p* = 0.514].

Despite the absence of major structural differences, finer morphometric analyses revealed that suicides differed significantly from controls with regards to DCX-IR process size. Suicides’ DCX-IR processes had both larger base diameters, particularly at 2nd and 3rd branch orders [*F*(1,969) = 20.12, *p* < 0.001; **Figure 3E**], and larger average diameters [*F*(1,966) = 19.47, *p* < 0.001; **Figure 3F**]. As a result, the overall volume of DCX-IR processes proximal to the soma was higher in suicides [*F*(1,873) = 14.16, *p* < 0.001; **Figure 3G**], whereas process volume decreased by ∼50% following the first branch point in controls.

**FIGURE 4:**
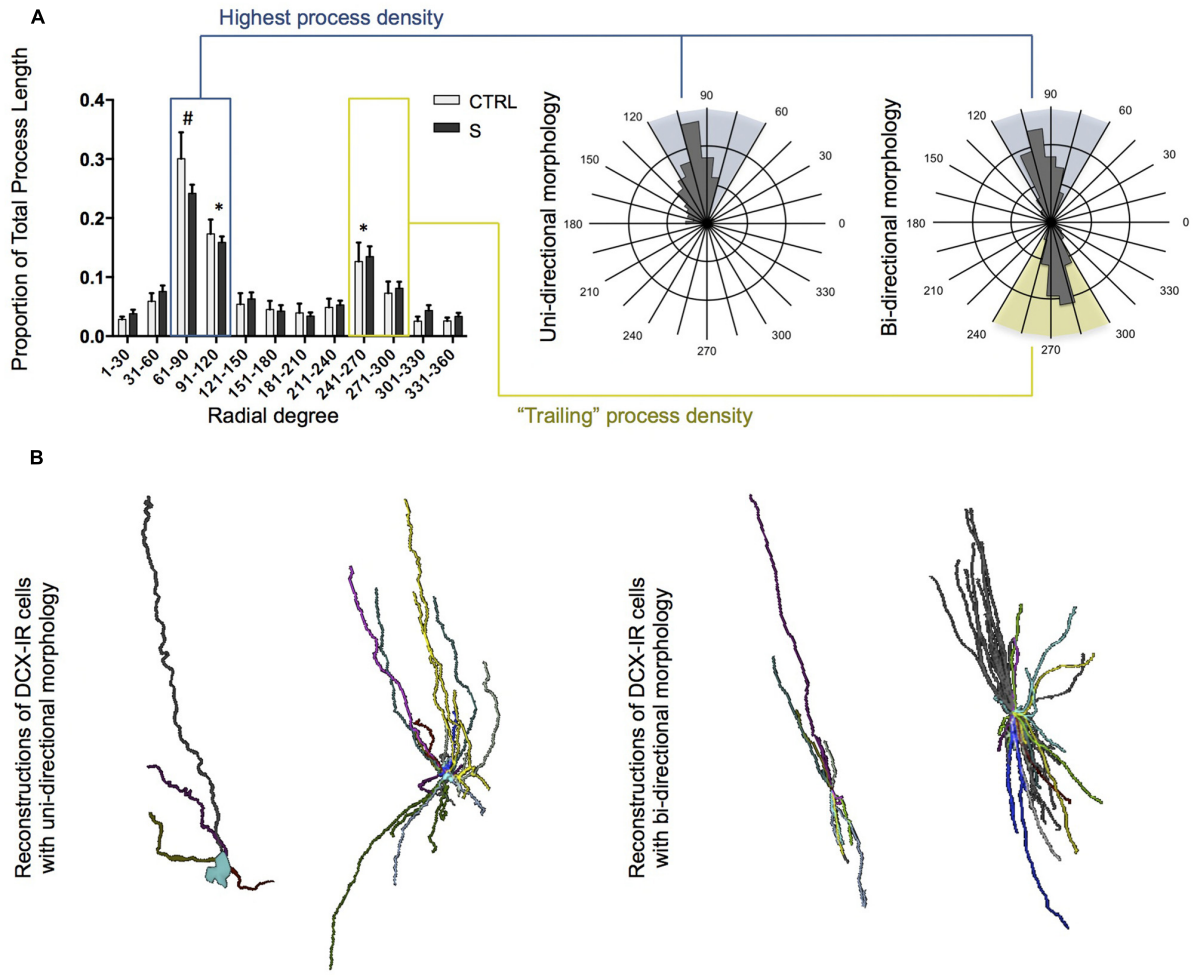
**Uni- and bi-directional structure of DCX-IR cells in the OT of suicides (S; *n* = 6 cells) and controls (CTRL; *n* = 19 cells). (A)** Fan-in analysis and sample radial plots of DCX-IR cells. When plotted, the proportion of total process length per radial degree revealed a bimodal distribution indicating that DCX-IR cells tend toward a uni- or bi-directional structure. Sample radial plots display Fan-in analyses for a uni- and bi-directional cells. Shaded areas correspond to the sextant of highest process density (blue shading and box) and the sextant opposite the region of highest process density (yellow shading and box). **(B)** Sample reconstructions of small/simple and large/complex DCX-IR cells in the human OT displaying uni- (left) and bi-directional (right) morphology. All data are plotted as means, and error bars depict SEM. # denotes a significant difference as compared to all other radial degrees; ^∗^ denotes a significant difference as compared to all other radial degrees except those also marked by an asterisk.

Notably, DCX-IR cells in the OT showed a strong tendency to align themselves rostrocaudally, with the majority of processes oriented in the same direction or, in some cases, grouped into bundles oriented in opposite directions. Fan-in analyses were used to quantitatively assess this phenomenon and confirmed that the radial distribution of DCX-IR multipolar cell processes was non-random. Instead, multipolar cells tended to display a uni- or bi-directional structure resulting in a bimodal Fan-in plot wherein the proportion of each cell’s total process length showed peaks separated by 180∘ (**Figure 4A**). Although there were no group differences, two-way ANOVA revealed a significant main effect of axial degree on proportion of process length [*F*(1,276) = 43.23, *p* < 0.001]. *Post hoc* tests confirmed that the process density was significantly higher within regions between 61∘–90∘ (which differed significantly from all other radial regions), 91∘–120∘, and 241∘–270∘ (which did not differ from one another, but did differ significantly from all other regions). Examples of both uni- and bi-directional morphologies were observed among small, simple cells as well as among larger, more complex DCX-IR cells (**Figure 4B**). In order to assess whether CTRL and S subjects differed with regards to the ratio of uni-directional to bi-directional DCX-IR multipolar cells present in the tract, we operationally defined DCX-IR cells with “bi-directional” morphology as those cells with a ratio of “highest process density” (blue sextant, **Figure 4A**) to “trailing process density” (yellow sextant, **Figure 4A**) of ≤2. Cells with ratios of >2 were considered to display uni-directional morphologies. Although suicides displayed a slightly higher proportion of cells with bi-directional morphologies than did controls (58 and 33%, respectively), this difference was not statistically significant.

### DCX-Immunoreactive Cell Numbers in the OT

**FIGURE 5:**
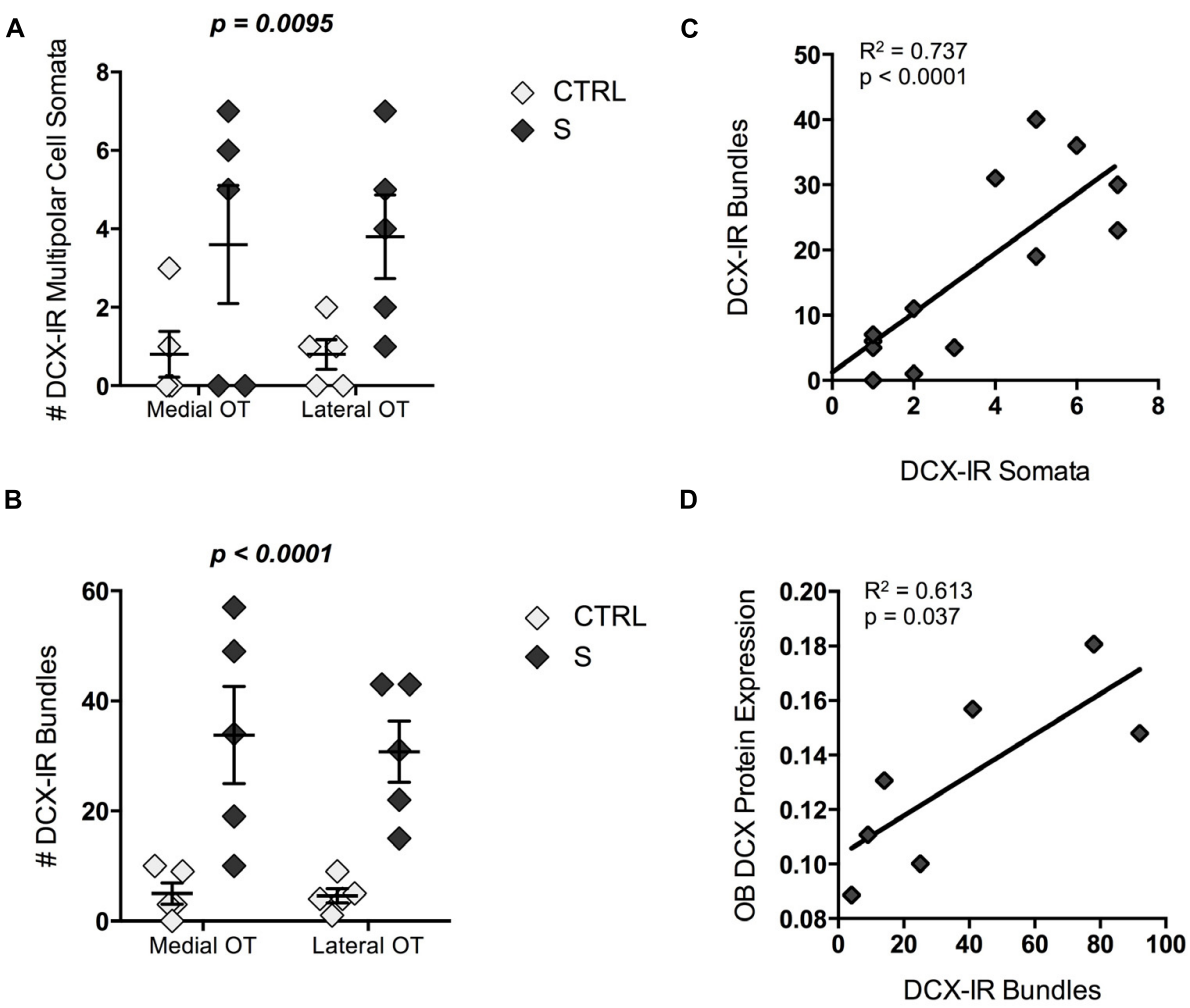
**Doublecortin-immunoreactive cells and process bundle number in the OT of individuals who died by suicide (S; *n* = 5) and controls (CTRLs; *n* = 5). (A,B)** Suicides had significantly more DCX-IR multipolar cell somata (*p* = 0.037) and process bundles (*p* = 0.001) in the OT than did CTRLs. **(C)** The total number of DCX-IR somata within the entire OT correlated positively and significantly with bundle number (*p* < 0.001). **(D)** DCX-IR bundles correlated, in turn, with DCX-immunoreactivity in the olfactory bulb as assessed by immunoblotting (*p* = 0.037). *P*-values above graphs represent significance of “group” main effects. All data are plotted as mean, and error bars depict SEM.

Perhaps the most striking characteristic of DCX-IR multipolar cells was their rarity. Controls possessed only one or two DCX-IR multipolar cells in the entire tract. By contrast, suicides had approximately four times as many cells per OT [*F*(1,18) = 5.097, *p* = 0.036; **Figure 5A**]. DCX-IR cells did not appear to be preferentially distributed within medial versus lateral OT regions. Likewise, DCX-IR process bundles were more common in the OT of individuals who died by suicide [*F*(1,18) = 14.33, *p* = 0.001; **Figure 5B**]. The number of DCX-IR somata correlated positively and significantly with the number of DCX-IR bundles in medial and lateral OT (*R*^2^ = 0.737, *p* < 0.001; **Figure 5C**), as did the total number of somata and bundles throughout the tract (*R*^2^ = 0.778, *p* < 0.001). Notably, the total number of DCX-IR bundles correlated, in turn, with protein expression in the OB (*R*^2^ = 0.613, *p* = 0.037; **Figure 5D**). Finally, when suicides were subdivided into medicated and unmedicated suicides and compared to CTRLs, DCX-IR bundle number was found to differ significantly between groups [*X*^2^(2,10) = 6.860, *p* = 0.032]. *Post hoc* tests revealed significantly more DCX-IR bundles in medicated suicides [*U*(7) = 15.00, *p* = 0.024], and a trend toward more bundles in the OT of unmedicated suicides [*U*(8) = 15.00, *p* = 0.051], as compared to CTRLs.

Taken together, these data suggest that the elevated expression of DCX observed in the OB of suicides may principally reflect an increase in the number of DCX-expressing multipolar cells. Furthermore, as with OB DCX levels, the number of DCX-IR cells does not appear to be affected by ADT.

## Discussion

The biological mechanisms underlying depression and antidepressant response are thought to involve altered cellular plasticity in fronto-limbic brain regions (Hercher et al., 2009a; Castren, 2013). In particular, strong evidence from rodent (Sahay and Hen, 2007; Anacker, 2014) and postmortem studies (Boldrini et al., 2009, 2012) has implicated adult hippocampal neurogenesis. Although there is considerable overlap in the molecular control of adult neurogenesis in the hippocampus and SVZ/OB, few investigations have explored the effects of stress and antidepressants on adult neurogenesis in the rodent SVZ/OB, and none have examined neurogenic markers in postmortem SVZ/OB samples from individuals having died during a depressive episode.

Doublecortin protein expression was found to be increased in both the OB and SVZ of unmedicated depressed suicides and, in the SVZ, this increase was strongly correlated with Sox2 protein expression. Previous reports have suggested that DCX and Sox2 may co-localize in neuronal precursors or early neuroblasts (Ashton et al., 2012; Batailler et al., 2014), findings in keeping with our results showing SVZ DCX-IR cells display morphologies consistent with both type B1 progenitor cells and migratory neuroblasts. In addition, Sox2 is widely expressed within the SVZ, and many immature and lineage-unrestricted cells, including proliferative cells and neuroblasts, express this protein (Mamber et al., 2013). Thus, our SVZ Sox2 expression data may reflect alterations in any of these subpopulations, a hypothesis supported by the strength of the correlation between Sox2 and DCX in this region. DCX levels in the OB and SVZ correlated positively and significantly, and our measures of relative DCX expression in these regions are consistent with previously published estimates of new neuroblast loss in the postnatal human RMS (Kam et al., 2009). Taken together, these findings raise the possibility that the same pool of cells is driving the elevated expression of DCX in both regions.

The DCX-IR cells described here were about four times more common in suicides and varied considerably in size, with the largest reconstructed cell having a volume ∼38 times that of the smallest. Nonetheless, their overall morphology, including their uni- or bi-directional structure and the branching pattern of their processes was remarkably uniform across DCX-IR cells of different sizes. With regards to group comparisons in DCX-IR cell structure, only process volume was found to differ, with suicides displaying a slight increase associated with larger process diameter. Thus, the principal difference we observed between controls and suicides was the number of DCX-IR multipolar cells present in the OT, not their morphology.

Although our examination of postmortem human tissue cannot directly reveal the source of the DCX-IR cells we identified in the OT, our data seem more consistent with the idea that these cells are the result of impaired migration and differentiation of SVZ-derived neuroblasts, than actively migrating neuroblasts or a specialized population of cells present in the OT since early development. Multipolar DCX-IR cells were extremely sparse and seemingly distributed at random throughout the OT; indeed, controls possessed only one or two in the entire tract. This rarity is at odds with the notion that they represent a distinct subpopulation of cells within the OT, though it does not rule out the possibility that a very small subset of developmentally derived neurons began, for whatever reason, to express DCX. Still, the heterogeneity in the size of these cells within both controls and suicides implies that they are not a single, fully developed neuronal subgroup with a shared phenotype, and the presence of bulbous dendritic tips common in differentiating neurons within the adult human hippocampus supports this hypothesis. OT DCX-IR cells also possessed a structural complexity at odds with the notion that they retained migratory potential.

**FIGURE 6:**
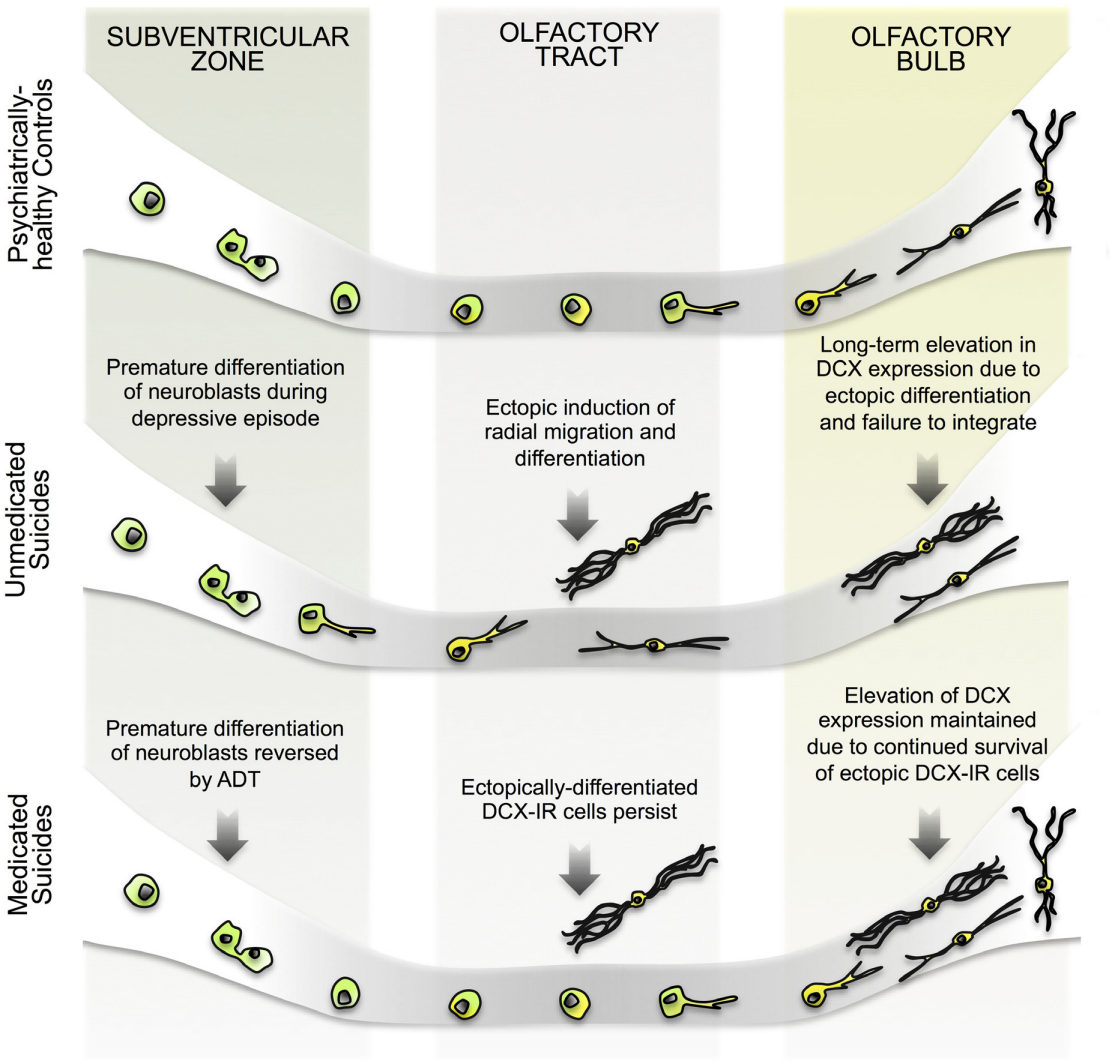
**Hypothetical model of changes in human SVZ and OB neurogenesis during depressive episodes. (Top)** In psychiatrically healthy subjects, Sox2-expressing progenitors in the SVZ proliferate to give rise to migratory neuroblasts which then migrate toward the OT. During tangential migration, neuroblasts begin the process of differentiation, perhaps extending a leading process, but retain an immature phenotype until they have reached the OB. Having reached the OB, neuroblasts migrate radially toward the granule cell layer and complete their differentiation, integrating into existing neuronal circuits. **(Middle)** In unmedicated suicides, DCX expression in the SVZ is elevated in the absence of any increase in proliferation due to the premature differentiation of neuroblasts. At this point of development, cells may co-express Sox2 and DCX, as previously reported (Ferri et al., 2004). This early differentiation of neuroblasts in turn impairs migration, and ectopically differentiating cells become trapped at points along their migratory path, including within the OB. In some cases, radial migration is elicited early, resulting in the presence of DCX-IR cells in outer layers of the OT. Alternately, cells fail to properly maintain directionality during tangential migration. In the absence of the appropriate environmental cues that instruct maturation and integration, cells differentiating ectopically may continue to express DCX longer than usual, resulting in persistently elevated DCX levels. **(Bottom)** Following ADT, induction of early differentiation of SVZ cells is halted, and DCX levels return to control levels as neuroblasts successfully migrate away from the region. In the OT and OB, however, previously differentiated cells persist alongside newer migrating cells. DCX levels in the OB are therefore elevated long-term, despite an absence of new ectopically differentiating cells. Future experiments may determine whether DCX expression in the OB eventually returns to control levels.

The positive correlation between SVZ and OB DCX levels observed among controls and suicides further suggests that DCX in the OB and SVZ are regulated in tandem, either by way of common upstream mechanisms or because one population is directly descended from the other. Considering our data as a whole, we suggest that the most parsimonious explanation for the presence of DCX-IR multipolar cells in the OT, then, is that these cells represent an aberration resulting from a migratory impairment among SVZ-derived neuroblasts (**Figure 6**). The apparent normalization of SVZ DCX expression following ADT additionally suggests that elevated DCX expression among suicides is a recent, and perhaps, partially reversible phenomenon. By extension, long-term antidepressants may likewise reduce the number of DCX-IR multipolar cells in the OT and normalize DCX expression in the OB.

Nonetheless, the precise source of the DCX-IR multipolar cells we observed in the human OT is unclear, and will remain so unless and until imaging techniques allowing the study of migration in the living human brain are developed. The possibility remains that DCX-IR cells in the SVZ and OB represent two distinct populations, and that ADT affects only those cells located within the SVZ. Alternately, only a subset of DCX-IR cells in the SVZ may be altered in depressed suicides, and thus normalized by ADT. If so, even long-term ADT may have little impact on DCX-IR multipolar cell number in the OT. Recent evidence suggests that SVZ-derived neuroblasts may continue to populate the human striatum throughout life (Ernst et al., 2014). Although these findings have been disputed (Wang et al., 2014), and more work will be required before conclusions regarding the neurogenic capacity of the striatum are reached, an examination of the effects of depression and ADT on DCX expression and neuroblast number in this region may help elucidate whether our findings reflect a general impairment of migration among SVZ-derived neuroblasts.

The precise mechanisms by which pharmacological antidepressants alter adult neurogenesis remain unclear but, in addition to modulating proliferation, recent work suggests that antidepressants alter the speed and efficiency of neuronal differentiation (Ferri et al., 2004; Wang et al., 2008). The capacity of antidepressant drugs to affect earlier, rather than later, phases of neurogenesis are in line with our findings that antidepressants appeared to normalize DCX expression in the SVZ while having no impact on OB DCX levels or DCX-IR cell number in the OT of suicides. If indeed DCX-IR multipolar cells within the OT are the result of a migrational deficit, there may be a critical window during which the developmental trajectory of immature neurons can be changed by antidepressants.

Depression-related olfactory deficits seem to persist after acute (∼14 days) ADT, whereas chronic (∼60 days) treatment appears to correct them (Pause et al., 2001, 2003). A similar temporal dissociation is frequently cited as evidence that hippocampal neurogenesis is a key component of antidepressant efficacy and improvement on hippocampal-dependent tasks (Dranovsky and Hen, 2006). However, adult neurogenesis in the hippocampus seems to occur at much higher rates in humans than it does in the OB (Sanai et al., 2011; Bergmann et al., 2012), and the rarity of the multipolar DCX-IR cells described here would seem to suggest that they are unlikely to be the principal cause of depression-associated olfactory deficits. Still, we cannot rule out the possibility that, given a sufficient number of ectopically differentiating cells accumulating along the several centimeters separating the SVZ from the OT, a migrational deficit such as we posit here might disrupt olfactory processing. Alternately, persistent increases in DCX expression among OB interneurons may alter OB function independently of any change in migration itself. An examination of OB immediate early gene activity may clarify the functional repercussions. Yet, without detailed information regarding the age of DCX-IR cells or how long subjects were taking antidepressants before they died, it will remain difficult to define the association between antidepressant medication, DCX cell number, and olfactory function. It is also important to note that the abovementioned studies by Pause et al. (2001, 2003), examined individuals for whom ADT successfully reversed depression severity. This was not the case for our own study, nor were we able to acquire sufficient samples from individuals in a remitted state to explore whether OB DCX expression and DCX-IR cell number is eventually normalized, as we hypothesize here.

To the best of our knowledge, DCX-IR multipolar cells have never been described in the rodent OT. Nor have migratory deficits or the ectopic differentiation of SVZ-derived neuroblasts been reported in animal models of depression. One possible explanation for this disparity may be that DCX-IR cell accumulation is inhibited by the relatively high rate of OB neurogenesis in most model animals whose SVZ-derived neurons display chain migration throughout life. OB neurogenesis in humans, by contrast, slows dramatically by adulthood (Wang et al., 2008; Kam et al., 2009), which may in turn deprive human neuroblasts of some of the cues that keep rodents’ migrating cells in line. For instance, neuronal precursors from mice deficient in PSA-NCAM, which is typically co-expressed by DCX-IR neuroblasts and facilitates migration, are nonetheless able to migrate when surrounded by PSA-NCAM expressing cells (Hu, 2000). Thus, in some cases, deficits that might otherwise impair migration may be undetectable given an otherwise permissive environment. Whether migration, differentiation, or the expressions of proteins involved in these processes are altered in adult-born hippocampal neuroblasts is also unclear. Since new neurons in the dentate gyrus need only travel a few cell widths before integrating into hippocampal circuits and the relatively high rate neurogenesis in the region, it is possible that whatever deficit contributes to the increased accumulation of DCX-IR cells in the OT have no effect on hippocampal cells. However, the possibility remains that their maturation may be impaired, given that signaling pathways regulating synaptic integration and neuroblast differentiation overlap. Further research will be needed to determine the extent of, and molecular contributors to, deficits in neuronal development in the depressed human brain.

The present study has limitations that include a small sample size, the heterogeneity of psychiatric diagnoses present in the suicide group, and the absence of samples from depressed patients not having died from suicide. Thus, although our data support the idea that DCX cell number and protein expression is elevated in the OB of individuals who died by suicide during the course of a depressive episode, regardless of their primary Axis I diagnosis, future work using larger group sizes may yet reveal a significant effect of Axis I disorder. Similarly, due to the rarity of the DCX-IR multipolar cells in the OT, we were only able to provide a preliminary characterization of these cells. Our 3-dimensional reconstructions revealed considerable variability in their size, which may have masked group differences that otherwise, would be apparent if greater numbers of cells had been available for investigation. Similarly, limited resources precluded the possibility of completing morphological analyses of DCX-IR cells in the OB itself. As a result, it remains unclear whether DCX-IR cell number or their distribution within the OB, are notably altered. Unfortunately, this scarcity is likely to remain a significant barrier to their study given the large number of human OB and OT samples that would be required to complete more detailed analyses.

## Conclusion

This study describes disruptions in the expression of DCX in the SVZ and OB of suicides that is associated with the accumulation of a rare population of DCX-IR multipolar cells in the OT. We hypothesize that this reflects a neuronal “failure to thrive” among suicides, and that this may be at least partially reversed by antidepressants. Future studies will be needed to determine whether this phenomenon is representative of alterations affecting similar neurodevelopmental processes elsewhere in the brain, and how antidepressant medications may mediate its reversal.

## Conflict of Interest Statement

The authors declare that the research was conducted in the absence of any commercial or financial relationships that could be construed as a potential conflict of interest.
