# Supplementary material for: Increased doublecortin (DCX) expression and incidence of DCX-immunoreactive multipolar cells in the subventricular zone-olfactory bulb system of suicides
Source: Front Neuroanat. 2015 Jun 1;9:74. doi: 10.3389/fnana.2015.00074 (PMC4450175; doi:10.3389/fnana.2015.00074)
Supplement: Supplementary file 1 [file Data_Sheet_1.DOCX]

***Supplementary Material***

**Evidence of impaired neuroblast migration from the subventricular zone of suicides**

**Marissa E. Maheu^1,2^, Julia Devorak^1^, Alexander Freibauer^1^, Maria Antonietta Davoli^1^, Gustavo Turecki^1,2,3^, Naguib Mechawar^*,1,2,3^**

^1^McGill Group for Suicide Studies, Douglas Mental Health University Institute, Montreal, Quebec, Canada

^2^McGill University, Integrated Program in Neuroscience, Department of Neurology and Neurosurgery, Montreal, Quebec, Canada

^3^McGill University, Department of Psychiatry, Montreal, Quebec, Canada

*** Correspondence: Dr. Naguib Mechawar, McGill Group for Suicide Studies, Douglas Mental Health University Institute, FBC Pavilion, 6875 LaSalle Blvd., Montreal, Quebec, Canada.**

**naguib.mechawar@mcgill.ca**

1. **Supplementary Figures and Tables**

## Supplementary Tables

**Supplementary Table 1. Characteristics and variability of doublecortin (DCX) immunoreactive cells in the olfactory tract of suicides (S) and controls (CTRL); n = 25 cells.**

## Supplementary Figures

**Supplementary Figure 1. Schematic representation of human subventricular (SVZ), olfactory bulb, and olfactory tract tissues used for immunohistochemistry (IHC), Nissl staining, and immunoblotting**. (**A**) Fresh-frozen samples of SVZ containing the anterior horn of the left lateral ventricle and corresponding to plates 7-12 of the *Atlas of the Human Brain* (Mai *et al.*, 2007) were collected and further dissected for IHC and protein extraction. SVZ samples used for immunoblotting contained the most ventral aspect of the left lateral ventricle, including the lateral wall, dissected to a thickness of ~2mm. IHC was performed using sections immediately dorsal to those collected for protein extraction and comprised the lateral wall extending toward the caudate nucleus. Frozen (left) olfactory bulbs were processed for immunoblotting, whereas tracts were processed for IHC. Because OB length varied between individuals, the OB was separated by cutting each sample at the rostral level where the OT began to expand into a characteristic bulbar structure (dashed line). Fixed (right) olfactory bulbs, including tract, were employed for Nissl staining and cell counting. (**B**) Venn diagram depicting overlap between subjects whose tissues were used for various experiments.

**Supplementary Figure 2. Granule cell layer neuronal, glial, and endothelial cell densities in the olfactory bulb (OB) of suicides (S) and non suicides (CTRL)**. Olfactory bulbs processed for Nissl staining were fixed in 10% formalin for 90 min at room temperature, then transferred into 15% and 30% sucrose solutions for 24 hours at 4°C prior to being sectioned sat 50 µm on a freezing microtome. Sagittal sections of OB with constant periodicity were collected, mounted on slides, labeled with 0.1% cresyl violet, dehydrated, and cover-slipped with Permount (Fisher Scientific, Ottawa, ON, Canada). (**A**) Neuronal (red arrow), glial (black arrow), and endothelial (white arrow) cell densities in the granule cell layer (GCL) were assessed using an Olympus BX51 microscope equipped with a motorized stage and a CX-9000 camera (MBF Bioscience, Williston, VT, USA). Cells were counted with a 60x (N.A. 1.35) oil immersion objective using an optical fractionator probe with the *Stereo Investigator* software (MBF Bioscience, Williston, VT, USA). Neurons, glia, and endothelial cells were identified using standard morphological criteria, including their size, shape, and staining pattern (i.e., presence of euchromatin or stained cytoplasm surrounding the nucleolus) (Garcia-Amado & Prensa, 2012; Hercher *et al.*, 2009b). All OB sections containing identifiable GCL were sampled. An average of 1500 cells were counted per subject by an experimenter blinded to group, and mean densities were calculated for GCL volume (calculated automatically using total area of outlined GCL region multiplied by the average tissue thickness after shrinkage as measured at each sampling site) and expressed as cells per mm^3^. (**B**-**D**) No significant differences in neuronal, glial, or endothelial cell densities were observed. Nissl cell counting subject information is presented in (**E**). Abbreviations: MDD, major depressive disorder; OCD, obsessive compulsive disorder; NSAID, non-steroidal anti-inflammatory drug; PMI, postmortem interval; SSRI, selective serotonin reuptake inhibitor. All data are plotted as means, and error bars depict standard error of the mean.

**Supplementary Figure 3. Relative expression of doublecortin (DCX) in the human subventricular zone (SVZ) and olfactory bulb (OB)**. (**A**) Immunoblotting of pooled SVZ or OB protein from a random subset of all subjects, along with tissues from additional subjects who did not meet criteria for inclusion in group comparisons, revealed that DCX expression was approximately 3-fold higher in the human SVZ than in the OB. (**B**) Two separate pooled samples from the SVZ and OB were used to replicate these results, and run in duplicate on two separate gels. These consisted of protein from a subset of controls (CTRL; n = 5) and, separately, protein from a subset of unmedicated depressed suicides (S; n = 3) which were selected based on the availability of tissue. Immunoblotting revealed that despite the presence of group differences in the absolute protein expression in both brain regions (see Figure 1), overall expression of DCX was approximately 3-fold higher in the SVZ than in the OB. All data are plotted as means, and error bars depict standard error of the mean.

**Supplementary Figure 4.** Expression of doublecortin (DCX) in (**A**) the human olfactory bulb (OB) and (**B**) subventricular zone (SVZ) of controls, unmedicated suicides (S) and antidepressant-treated suicides (S + ADT) with various primary Axis I diagnoses. Colours denote the following primary Axis I diagnoses: white, no diagnosis; black, depression; gray, psychotic disorder; blue, Obsessive Compulsive Disorder; yellow, Bipolar Disorder; green, substance dependence. All data are plotted as means, and error bars depict standard error of the mean.
